# Influence of irrigation needle type on apical cleaning efficacy in endodontic treatment - An *In Vivo* study

**DOI:** 10.6026/973206300212373

**Published:** 2025-08-31

**Authors:** Debkant Jena, Mattapudi Basavaiah Babu, Bhavana Soni, Ashtha Arya, Amit Kumar Joseph, Shreya Charudutt Patil

**Affiliations:** 1 Department of Conservative Dentistry & Endodontics, Kalinga Institute of Dental Sciences, KIIT University, Bhubaneswar, India; 2 Department of Conservative Dentistry and Endodontics, Anil Neerukonda Institute of Dental Sciences, Tagarapuvalasa, Visakhapatnam andhra Pradesh, India; 3 Department of Oral and Maxillofacial Pathology & Microbiology, Surendra Dental College and Research Institute, Sri Ganganagar, Rajasthan, India; 4 Department of Conservative Dentistry and Endodontics, SGT Dental College, Hospital and Research Institute, SGT University, Gurugram, Haryana, India; 5 Department of Oral and Maxillofacial Pathology and Microbiology, Teerthanker Mahaveer University, Moradabad Uttar Pradesh, India; 6 Department of Conservative dentistry and Endodontics, Pandit Deendayal Upadhyay Dental College, Solapur, Maharashtra, India

**Keywords:** Endodontic irrigation, irrigation needle, root canal treatment, apical debris removal, side-vented needle, open-ended needle, double-side vented needle, sodium hypochlorite, root canal cleanliness, irrigant delivery system, canal disinfection

## Abstract

The impact of various irrigation needles on apical cleaning during endodontic treatment was assessed in this *in vivo* investigation. The use of open-ended, side-vented or double-side vented needles determined the patients' grouping. Apical cleanliness was evaluated under a microscope after standard irrigation with sodium hypochlorite was completed. Open-ended needles performed the worst in terms of cleaning effectiveness, while double-side vented needles were the most effective. The findings imply that needle design has a major impact on the removal of apical debris and ought to be carefully taken into account in clinical practice.

## Background:

A key component of successful endodontic therapy is efficient irrigation. Given its intricate structure, lateral canals, smear layer and biofilms, the entire root canal system especially the apical third cannot be sufficiently cleaned by mechanical instrumentation alone [1, 2]. Irrigants like sodium hypochlorite (NaOCl) are essential for breaking down organic materials and getting rid of bacteria, but how they are applied and the equipment they use greatly affect how effective they are [3, 4]. The flow pattern, apical penetration and subsequent debris removal are all significantly influenced by the irrigation needle's design. According to studies, closed-ended and side-vented needles improve irrigant distribution in the canal space while lowering the chance of apical extrusion [5, 6]. Conversely, open-ended needles have a greater chance of apical extrusion and less efficient lateral flow, but they might permit deeper penetration [7]. In clinical practice, optimal apical cleaning is still difficult to achieve despite improvements in irrigant formulations and delivery methods. To find designs that maximise cleaning effectiveness while reducing complications, comparative evaluations of different types of needles are necessary. Therefore, it is of interest to assess and contrast the effectiveness of various irrigation needle designs more especially, open-ended, side-vented and double-side vented needles in terms of apical cleaning during routine endodontic therapy.

## Methodology:

This *in vivo* comparative study was conducted over a period of one year in the Department of Conservative Dentistry and Endodontics to evaluate the apical cleaning efficacy of different irrigation needle designs during root canal treatment. A total of 90 patients, each requiring non-surgical endodontic therapy on single-rooted teeth, were included based on clinical and radiographic criteria. The sample size was determined using statistical methods appropriate for detecting a medium effect size with a significance level of 0.05 and 80% power. Patients were randomly allocated into three equal groups (n = 30) based on the type of irrigation needle used: open-ended, side-vented and double-side vented. All patients were between 18 and 50 years of age, systemically healthy and presented with teeth that had no previous endodontic treatment or anatomical complications such as resorption or open apices. Standardized root canal treatment was performed using rotary NiTi instrumentation (ProTaper Universal), with working length determined using an apex locator and confirmed radiographically. Irrigation was carried out using 3% sodium hypochlorite with a total volume of 5 mL between each file and a final rinse with 5 mL saline, maintaining a needle insertion depth of 1 mm short of working length. Each group received irrigation through its assigned needle under consistent conditions. After obturation, the roots were longitudinally sectioned and examined under a stereomicroscope at 20x magnification to assess apical cleanliness using a debris scoring system ranging from score 1 (clean) to score 4 (heavy debris). Data were analyzed using SPSS software and statistical comparisons among groups were made using the Kruskal-Wallis test followed post hoc analysis. A p-value of less than 0.05 was considered statistically significant.

## Results:

The study demonstrated that the type of irrigation needle significantly influenced apical cleaning efficacy. The distribution of cleaning scores showed that the double-side vented needle group had the highest number of clean canals, while the open-ended group exhibited more moderate to heavy debris cases (Table 1 - see PDF). When analyzed as percentages, 50% of cases in the double-side vented group were debris-free, compared to 33.3% in the side-vented group and only 16.7% in the open-ended group (Table 2 - see PDF). The mean apical debris score was lowest in the double-side vented group (1.7), followed by the side-vented group (2.0) and highest in the open-ended group (2.5), indicating superior cleaning efficacy with lateral-venting designs (Table 3 - see PDF). These differences highlight the clinical relevance of needle design in achieving effective apical debridement during endodontic irrigation. The distribution of apical cleaning scores across the three groups highlights significant differences in debridement efficacy. Group C (double-side vented) showed the highest proportion of "Score 1 - Clean" cases, while Group A (open-ended) had a more even distribution across all score categories, including a higher proportion of heavy debris (Score 4) (Table 1 - see PDF). When expressed as percentages, 50% of cases in Group C were classified as clean compared to only 16.7% in Group A, indicating superior performance by the double-side vented needle. Furthermore, Group A showed the highest percentage (16.7%) of heavy debris cases, while Group C showed the least (3.3%) (Table 2 - see PDF). The mean apical cleaning score was lowest in Group C (1.7), followed by Group B (2.0) and highest in Group A (2.5), confirming that double-side vented needles provided significantly better cleaning efficacy than the other two designs (Table 3 - see PDF).

## Discussion:

The impact of various irrigation needle designs on the effectiveness of apical cleaning during endodontic treatment was evaluated in the current *in vivo* study. The findings showed that, in comparison to side-vented and open-ended needles, double-side vented needles removed more debris in the apical third. These results highlight how crucial needle design is for improving irrigant penetration and efficacy, especially in the root canal system's hardest-to-clean areas. Because of the intricate canal anatomy, restricted irrigant access and extrusion risk, cleaning the apical third continues to be one of the most difficult tasks in endodontic therapy. Better lateral dispersion and less apical vapour lock were probably encouraged by the use of double-side vented needles, which enhanced debris flushing. These results are in line with earlier research that demonstrated that needle tip design and placement depth have a significant impact on apical fluid dynamics, which in turn affects shear forces on canal walls and irrigant exchange [8]. Furthermore, the creation of turbulent flow and decreased apical pressure are responsible for the enhanced performance of side-vented and double-side vented needles, which improve irrigation safety and effectiveness. This finding has been corroborated by computational fluid dynamics studies, which demonstrate that side-venting configurations minimise apical extrusion while preserving efficient irrigant agitation [9]. Contrarily, the open-ended needle creates a unidirectional stream with little lateral coverage and a greater chance of periapical extrusion, which may result in less than ideal cleaning and more complications after surgery [10]. The *in vivo* findings of Gopikrishna *et al.* emphasize that needle gauge significantly influences irrigant flow rate, with larger-gauge needles (26G) delivering higher flow compared to narrower ones (30G). This has direct clinical implications, as increased irrigant volume and flow may enhance apical cleaning efficacy, although the risk of extrusion must also be considered [11]. In order to maximise treatment results, this study adds credence to an increasing amount of data that supports advancements in irrigation methods and delivery systems. Double-side vented needles may help lower failure rates and improve long-term prognosis by reducing debris retention and increasing chemical contact with canal walls. Future research could compare results with negative-pressure irrigation systems and investigate their advantages in multi-rooted teeth in more detail. However, this study emphasises that even minor adjustments to delivery methods, like choosing the right needle, can significantly enhance endodontic clinical outcomes [12]. Rajeswari *et al.* (2025) demonstrated that irrigation needle design significantly affects apical debris removal efficiency, with side-vented needles providing better cleaning compared to open-ended designs. This finding supports optimizing needle selection to enhance endodontic irrigation outcomes [13].

## Conclusion:

The design of irrigation needles significantly influences apical cleaning efficacy during root canal therapy. Double-side vented needles demonstrated superior debridement of the apical third compared to side-vented and open-ended designs. Further studies with larger sample sizes and advanced imaging are recommended to validate these findings.

## References

[1] Peters OA. (2004). J Endod..

[2] Siqueira JF-Jr, Roças IN. (2008). J Endod..

[3] Mohammadi Z, Shalavi S. (2012). Saudi Dent J..

[4] Zehnder M. (2006). J Endod..

[5] Boutsioukis C (2013). Int Endod J..

[6] Nielsen BA, Baumgartner JC. (2007). J Endod..

[7] Abou-Rass M, Piccinino MV. (1982). J Endod..

[8] Boutsioukis C (2010). J Endod..

[9] Jiang LM (2010). J Endod..

[10] Sedgley CM (2005). Int Endod J..

[11] Gopikrishna V (2016). J Conserv Dent..

[12] Desai P, Himel V. (2009). J Endod..

[13] Rajeswari PR (2025). J Conserv Dent Endod..

